# Supplementary material for: Reduced Graphene Oxide Modified Nitrogen-Doped Chitosan Carbon Fiber with Excellent Electromagnetic Wave Absorbing Performance
Source: Nanomaterials (Basel). 2024 Mar 27;14(7):587. doi: 10.3390/nano14070587 (PMC11013263; doi:10.3390/nano14070587)
Supplement: Supplementary file 1 [file nanomaterials-14-00587-s001.zip › nanomaterials-2900366-supplementary.pdf]

Supporting information

Reduced Graphene Oxide Modified Nitrogen-doped Chitosan Carbon Fiber with Excellent Electromagnetic Wave Absorbing Performance

Mengyao Guo[1], Ming Lin[1], Jingwei Xu[1], Yongjiao Pan[1], Chen Ma *[1] and Guohua Chen *[1]

[1] College of Materials Science and Engineering, Huaqiao University, Xiamen, 361021, China; 1614111009@stu.hqu.edu.cn (M. G.); 21014081077@stu.hqu.edu.cn (M. L.); 23011081017@stu.hqu.edu.cn (J. X.); 2014141019@stu.hqu.edu.cn (Y. P.)

* Correspondence: chenma@hqu.edu.cn (C. M.); hdcgh@hqu.edu.cn (G. C.)

1. Vector network analyzer (VNA) introduction

VNA is a specialized instrument for fast and automatic measurement of component, network, and system characteristic parameters, and automatic correction of errors. It is a special instrument that can realize relatively high-precision measurement results and automatically complete the conversion from one network parameter to another network parameter. VNA can obtain the amplitude and phase of the measured parameters at the same time, comprehensively measure the network scattering parameters (S-parameters), and quickly measure the amplitude and phase Angle of all S-parameters in single-port and dual-port networks. As shown in Fig.1, the VNA is mainly composed of four parts: synthetic signal source (excitation source), signal separation device (S-parameter measurement device), amplitude-phase receiving device, and processing display system. When the VNA works, signals of different frequencies are first generated by the synthetic signal source and divided into two channels through the signal separation circuit, one of which is the reference signal R and the other is the excitation signal of the Device Under Test (DUT). After the excitation signal passes through the DUT, the reflected signal A and the transmitted signal B are generated, which are extracted by the directional coupler in the signal separation device, and then the RF signals R, A, and B are processed by frequency conversion in the amplitude-phase receiver to generate the IF signal. The system adopts phase-lock technology. The amplitude and phase information of the measured network contained in the IF signal is converted to a digital signal by an A/D analog-to-digital converter, extracted by the digital signal processor, and the S-parameter of the measured network is calculated by ratio calculation. Finally, the test results are displayed on the screen in the form of graphs or data. Therefore, the reflection loss obtained in this way is the data that takes into account the influence of the electromagnetic wave transmission coefficient.

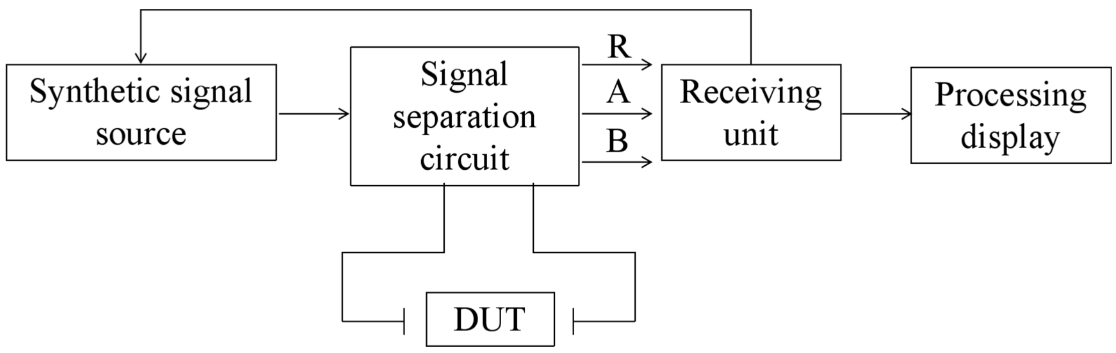

Fig.S1 Schematic diagram of VNA.

2. Electromagnetic parameters and absorption performance tests

To study the electromagnetic properties of carbon fibers at different carbonization temperatures, an appropriate network analyzer was used to test and analyze the above samples. The sample was shear crushed, mixed with paraffin wax in different proportions, and pressed into a homemade coaxial mold with an outer diameter of 7.00 mm, an inner diameter of 3.04 mm, and a thickness of 2.00-3.50 mm (as shown in Fig. 2(a)) for analysis and test.

At this stage, absorbing materials are mainly used mobile phone communication signals, wifi LAN (the operating frequencies is 2.4 GHz and 5.0 GHz), synchronous satellites (2.4 GHz and 2.6 GHz) and radar systems (L-band, 1-2 GHz; S-band, 2-4 GHz; C-band, 4-8 GHz; X-band, 8-12 GHz; Ku-band, 12-18 GHz), all of them are concentrated in 2-18 GHz. Therefore, in this work, the 2-18 GHz band is used as the test interval to measure the wave absorption performance of the sample.

The electromagnetic parameters of samples in the 2-18 GHz frequency range were measured by a VNA (Keysight Agilent E5071C) under an alternating electric field. The relative complex permittivity (real part ε' and imaginary part ε'') and the relative complex permeability (real part μ' and imaginary part μ'') of the sample were

measured by a VNA in the frequency range 2-18 GHz. The microwave absorption performance of the single-layer absorbing coating based on the ideal conductor was analyzed with the theoretical simulation of the transmission line. The variation of electromagnetic wave reflection loss [*RL*(dB)] value with *f* of samples with different thicknesses is simulated. The *RL* value was calculated by the normalized formula[1].

$$RL = 20\lg\left|\frac{Z_{in}-1}{Z_{in}+1}\right|$$

$$Z_{in} = \left(\frac{\varepsilon}{\mu}\right)^{1/2}\tanh\left[j\left(\frac{2\pi fd}{c}\right)(\varepsilon\mu)^{1/2}\right]$$

Where, Z_{in} is the input impedance at the interface between free space and the wave-absorbing coating, ε (ε'-j ε'') and μ (μ'-j μ'') are the relative complex permittivity and permeability of the wave-absorbing material, *d* is the thickness of the material, *f* is the frequency of the incident electromagnetic wave, and *c* is the speed of light. Through the comparative analysis of electromagnetic parameters between different samples, the changes in absorbing properties of materials under different sampling conditions and different frequencies were studied.

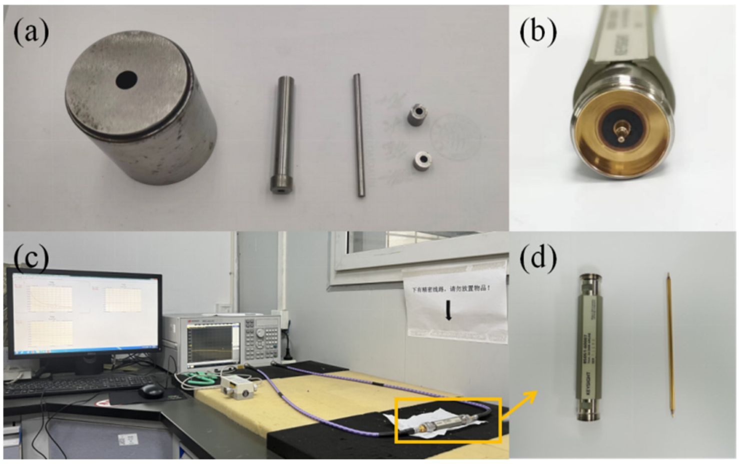

Fig.S2 Preparation of PCNF-wax composite samples and test details: (a) Coaxial ring mold for testing sample, (b) ring sample loading method, (c) electromagnetic

parameters measurement in the frequency range 2-18 GHz by VNA, (d) Keysight and the gold needle.

References

[1] Zhang X F, Li Y X, Liu R G, et al. High-Magnetization FeCo Nanochains with Ultrathin Interfacial Gaps for Broadband Electromagnetic Wave Absorption at Gigahertz. ACS Applied Materials & Interfaces, 2016, 8(5): 3494-3498.
